# Safety Impact Analysis Considering Physical Failures and Cyber-Attacks for Mechanically Pumped Loop Systems (MPLs)

**DOI:** 10.3390/s22134780

**Published:** 2022-06-24

**Authors:** Wenbo Wu, Lu Zhang, Hongyong Fu, Ke Wang, Xuzhi Li

**Affiliations:** 1 Technology and Engineering Center for Space Utilization, Chinese Academy of Sciences, Beijing 100094, China; wuwenbo@csu.ac.cn (W.W.); zhanglu@csu.ac.cn (L.Z.); fuhongyong@csu.ac.cn (H.F.); wangke@csu.ac.cn (K.W.); 2 The Key Laboratory of Space Utilization, 9 Dengzhuang South Road, Haidian Distirct, Beijing 100094, China

**Keywords:** safety impact analysis, mechanically pumped loop systems, physical failure, cyber-attack

## Abstract

As complex systems composed of physical and cyber components, mechanically pumped loop systems (MPLs) are vulnerable to both passive threats (e.g., physical failures) and active threats such as cyber-attacks launched on the network control systems. The impact of the aforementioned two threats on MPL operations is yet unknown, and there is no practical way to evaluate their severity. To assess the severity of the impact of physical failures and cyber-attacks on MPLs, a safety impact analysis framework based on Elman Neural Network (ENN) observers and the Gaussian Mixture Model (GMM) algorithm is suggested. The framework discusses three common attack and failure modes: sensor hard failure that occurs suddenly, sensor soft failure that occurs gradually over time, and denial-of-service (DoS) attacks that prevent communication between the controller and valve. Both sensor failures and DoS attacks render the system unsafe, according to simulation data. In comparison to DoS attacks, however, sensor failures, particularly soft failures, inflict the greatest harm to the MPLs. Furthermore, sensors engaged in global control, rather than those involved in local control, need additional protection.

## 1. Introduction

The China’s Tiangong space station, a massive on-orbit research laboratory, has played a critical role in enabling humans to conduct long-term microgravity scientific experiments [1]. China launched Tianhe, the first core module, in May 2021, which could serve as the Tiangong space station’s administration and control center. Wentian and Mengtian, the other two experimental modules, are set to debut in 2022. Experiment racks installed in the Tiangong space station will be used to conduct hundreds of experiments on new materials, space life science, fluid physics and basic physics. The precise thermal control requirement of racks is growing more stringent as the requirements of space experiments become more sophisticated. To fulfill the demands of precise thermal management, a new type of mechanically pumped loop system (MPL) has been created to offer thermal conditioning for numerous experiment racks. The heat produced by the payload is efficiently collected and discharged to the exterior space through a circulating cooling liquid and heat exchanger. This cooling system offers superior benefits in temperature control, long-distance heat transfer, huge heat transfer and stability when compared to typical temperature control systems such as cold pipes [2]. Despite their many benefits, MPL research is still in its infancy. Some critical concerns, particularly those concerning reliability and safety, must be addressed appropriately. When the system fails and no appropriate protective measures are taken, the functionality of different rack components will be harmed, and experiments will fail due to lack of heat dissipation. Poor heat dissipation increases the likelihood of the failure of all components of the experimental rack [3]. It may also cause casualties, especially for manned space stations; therefore, the ramifications are severe. To boost the system’s safety, it is vital to assess the effects of components failures and cyber-attacks on MPLs and create effective preventive measures.

The component-level failures and related diagnosis techniques have been extensively investigated. As one of the most vital components, data-driven methods [4,5], knowledge-based methods [6] and intelligent methods [7,8,9] are used for pump fault detection. Bhandari et al. [10] proposed a quick detection method of flow interruption owing to pump failure of a pumped fluid heat rejection system. By measuring the temperature change near the circulating pump, the fault diagnosis can be realized much earlier. Zhen Sun et al. [11] put forward a self-adaptive diagnosis method for heat pump systems based on a residual data and data scaling strategy, which adapts varying severity diagnosis under the condition that the training data derives from a single severity level. Zhonghai MA [12] used a nonlinear unknown input observer (NUIO) to diagnose the pump’s three failure modes: leakage, fatigue damage, and aging. The majority of sensors in MPLs are situated in harsh environments (such as high temperatures over 50 °C and underwater), rendering them susceptible to fouling and damage, which will impact the system’s precision, stability and reliability [13]. The early identification of sensor faults is critical for making corrective actions to mitigate the impact [14]. Linfeng Gou [15] established an intelligent approach that combines time-frequency analysis and CNN methodology to transform the signal recognition problem into an image recognition problem for effective sensor fault diagnosis. The method is cheap and simple to use compared to other quantitative model-based methods that demand complex mathematical models of the systems. D. G. Down [16] developed an observer-based fault diagnosis method to detect and isolate sensor faults. In addition to pump and sensor failure, heat exchanger faults [17], valve faults [18], and refrigerant charge faults [19] are also discussed. While prior research has explored component-level physical failures in depth, few have considered how component-level failures affect MPLs system function from a system-level viewpoint

Despite the significance of how cyber-attacks could affect MPLs performance, few studies have examined the impact. Fan Zhang [20] simulated five cyber-attacks, including man-in-the-middle (MITM), denial of service (DoS), data exfiltration, data manipulation, and fake data injection. Wei Wang [21] proposed a security margin calculation approach for prioritizing cyber threats in nuclear power plant thermal control systems. ShixingDing [22] provided a complete analytical methodology integrating optimization dispatch and simulation for cyber-attacks on heating systems, analyzing the influence of three particular cyber-attack models on system security. Kaveh Paridari [23] developed a groundbreaking cyber-physical security system that incorporates an analytics tool that can perform impact analysis whenever an attack is detected. However, the aforementioned study focused primarily on cyber-attacks and their effects on the cyber part of communication network systems. The impact on the physical part, such as the pipe system, was ignored.

The following is an overview of the research gaps in terms of modeling and evaluating physical failure and cyber-attacks on MPLs:

- How component-level failures that influence the functioning of the whole system require more research, and an appropriate way to assess the severity of the damage at the system-level should be suggested.
- Traditional safety analysis methods treat the cyber and physical components of MPLs separately. However, the coupling effects of cyber-attacks on physical components need more research.
- A co-simulation model capable of concurrently simulating physical failures and cyber-attacks is still required.

In response to the aforementioned research gaps, the following contributions are made in this paper: On the basis of AMESim [24] and Simulink software, a flexible and extensible simulation model is built. The model can accurately simulate MPLs and their network-based control system. By modifying the model’s parameters, various failures and cyber-attacks can be simulated, and the effect of these threats on the MPLs can be observed. A quantitative safety impact analysis method based on safety baseline and Mahalanobis distance [25] is proposed. Elman neural network (ENN) is employed as the system observer to extract the residuals of safe state and unsafe state by utilizing normal and abnormal data. At last, the migration index defining the safety of MPLs is calculated.

The paper is structured as follows: in Section 2, the structure of MPLs and simulation model, which are the basis for subsequent analysis method, are described. Typical physical failures, cyber-attacks, and their mathematical models are summarized. In Section 3, several proposed methods, including fault observer and Gaussian Mixture Model (GMM), are described in depth. In Section 4, we demonstrate the effectiveness of the proposed approaches through three examples. Finally, Section 5 concludes this paper.

## 2. MPLs Description and Simulation Model

**Figure 1:**
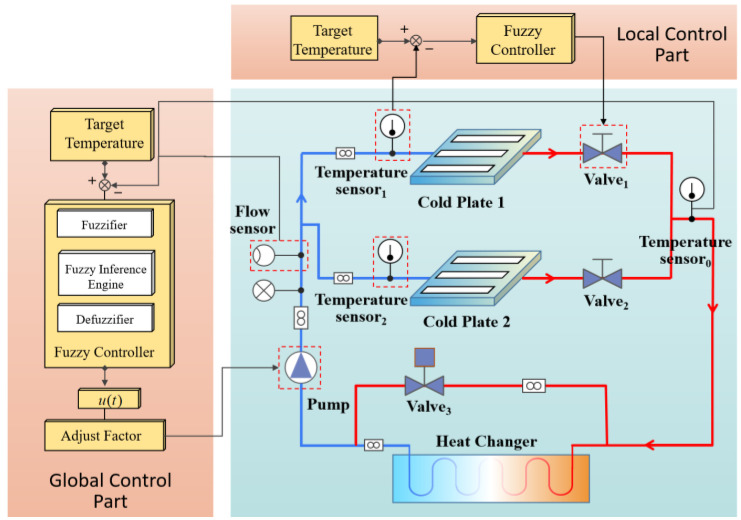
The typical structure of a MPLs.

The MPLs are intended to keep experimental payloads within their defined temperature range. Figure 1 depicts the arrangement of MPLs in an experiment rack. As shown in the figure, typical MPLs contain the pump, cold plate, sensor, valve and control system. The pump circulates cooling water across the cold plate to recover waste heat. After collecting the waste heat, the cooling water exports it to outer space through the heat exchanger and then returns to the cold plate to complete a closed cycle. The pump’s primary function is to supply a steady flow of cooling water to the MPLs, while the cold plate absorbs and dissipates the payload’s heat to maintain an optimal suitable temperature range. The electronic regulating valve is used to further adjust the flow of the cold plate on each branch to cope with the varying thermal load of the payload. The control system is designed to maintain the payload within an optimal temperature range, and it may be modified when the payload’s heat output fluctuates. The control system has two control layers: (1) using the local hierarchy, the flow is regulated at each branch valve, hence controlling the output temperature at each branch. (2) Using the global hierarchy, the pump speed is modified in order to regulate fluid temperature and flow at the main road outlet. The control cycle of the local controller is shorter than that of the global controller to ensure that cold plate temperature is more accurately controlled.

**Figure 2:**
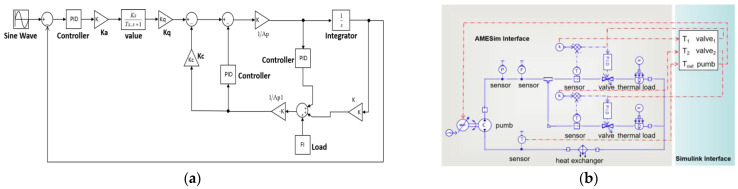
Co-simulation model (**a**) Simulink part; (**b**) AMESim part.

In order to achieve effective simulation of failures and cyber-attacks, a co-simulation model including both mechanical and control system parts is constructed in this research. Figure 2a depicts the control part of the MPLs, which is developed in a Simulink environment; Figure 2b depicts the mechanical part of the MPLs including pumps, flow valves, and sensors, which is modeled in AEMSim software. In AMESim, the mechanical component of MPLs is transformed to a Simulink S-Function, which can subsequently be imported into Simulink. Table 1 displays the control variables settings.

The threats of MPLs can be divided into two categories: physical failures and cyber-attacks. As a typical physical failure, sensors’ failure will change the output value, resulting in inaccurate measurement information. Especially when the sensor is used as the input of the control strategy, it will destabilize the system. According to [26,27], sensor failure types can be categorized into hard and soft failure. Soft failures are those that arise gradually, such as degradation, while hard failures come abruptly, such as bias and open circuit. In addition to physical failures, MPLs are vulnerable to malicious human-initiated cyber-attacks such as deception, denial of service (DoS attack), replay, etc. [28]. Cyber-attacks occur primarily during the communication between a sensor and a controller or between a controller and an actuator. Once the attack occurs, it will prevent actuators and controllers from receiving the latest data or sending information from sensors to controllers. Typical modes for cyber-attacks include delay and denial of service. In this study, we assume that once the network is paralyzed and the controller cannot send any signals, the actuators will only use the value before the DoS attack.

By tuning the parameters of related dynamic models, failures and attacks can be introduced into the simulation model, e.g., the sensor hard failures are introduced by adding a fixed bias [29]. A detailed introduction to the failure modeling and mathematical formulations are shown in Table 2, where $\hat{y}(t)$ and y(t) are the output value of sensors in normal state or fault state, respectively, $\overset{⌢}{u}(t)$ and u(t) are the control command values sent by controller to actuator under normal and fault conditions, respectively, and *A* is the failure time.

## 3. Imp3. Impact Analysis Based on Observer and GMM

In order to realize the safety impact analysis of MPLs, we proposed the method based on fault observer and GMM. The method contains two parts.

Part 1 is the creation of safe-state baseline. In this part, firstly, an observer is constructed using an Elman Neural Network (ENN) to estimate the normal output of the system. The residual errors are compared with normal and actual output. Then, the features are obtained based on Principal Component Analysis (PCA) [30]. Finally, the baseline GMM containing the optimized probability distribution function (PDF) is obtained.

**Figure 3:**
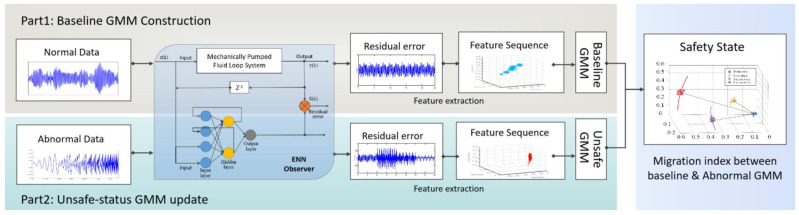
Framework of the proposed methodology.

Part 2, for the abnormal data obtained through the fault simulation process, the residual error and corresponding features can be obtained using the method described in part 1. When the PDF of the unsafe state GMM is obtained, the migration index (MI) is used to quantify the distance from the unsafe state GMM to the safe state GMM. When there are no faults or attacks in the MPLs, the PDFs of the unsafe state GMM and the safe state GMM are overlapped, and the value of MI is at its lowest value. When a failure or attack occurs, the PDF of the insecure state GMM will change, causing a corresponding change in its MI value. Thus, the MI can reflect the safety state of the MPLs. The greater the MI, the less secure the system. Figure 3 depicts the structure of the safety impact analysis methodology.

The method can be summarized in the following steps:

Step1. Using ENN, observers calculate the residual error of MPLs in safe and unsafe states, respectively.
Step2. Obtain the features of residual error-based PCA.
Step3. Apply GMM to estimate the PDF based on step 2.
Step4. Calculate MI which represents the system safety state.

### 3.1. Observer Based on Elman Neural Network

MPLs are non-stationary and non-linear due to fluid compressibility, friction, pump pulsation, and other non-linear mechanism characteristics. Therefore, the faults of MPLs and their influence mechanisms are very complex, and it is difficult to establish a fault observer based on the state space equation. As a time-delay feedback network, ENN is utilized to establish the fault observer to obtain residual information owing to its superior dynamic performance and strong nonlinear mapping capability.

ENN contains four layers: context, hidden, input and output. The input layer node serves as the channel entry to accept training sample or test sample data and transfer it to the hidden layer. The hidden layer contains a transfer function, and the weight and threshold in the node of the layer are used to calculate the data in the input layer. The feedback layer and the hidden layer establish a local feedback mechanism, receive the output of the hidden layer and feed it back to the hidden layer to form a closed-loop transmission of data, therefore realizing the delayed memory and dynamic learning functions of the neural network information. ENN can carry out accurate modeling through its feedback mechanism. Even if the mathematical model of the system is unknown, input and output sample data are sufficient.

The state space equation of ENN is shown in Equations (1)–(3) [31].

(1) $$x(k)=F(\omega^{1}x_{c}(k)+\omega^{2}u(k-1)+b_{1})$$

(2) $$x_{c}(k)=x(k-1)$$

(3) $$y(k)=G(\omega^{3}x(k)+b_{2})$$

where *x*(*k*) and *x_c_*(*k*) denote the output vectors of hidden layers and context layers, respectively; *w*^1^, *w*^2^ are the weight of context layers and the weight of input layers; *w*^3^ is weights matrix between hidden layer and output layer; *b*_1_ and *b*_2_ are the threshold vectors for the hidden and output layers. *F*(*x*) and *G*(*x*) represent the transfer function of neurons in hidden layer and output layer, respectively.

The residual error can be defined as:

(4) $$\varepsilon (k)=y_{r}(k)-{\overset{⌢}{y}}_{r}(k)$$

where ${\overset{⌢}{y}}_{r}(k)$ denotes the estimated output generated by the ENN observer, while *y_r_(k)* denotes the actual output.

In safety state, the residual error is close to zero and only affected by noise and modeling error. We define the residual error in this case as the initial benchmark. When a fault or attack occurs, the residual error will deviate from the benchmark.

### 3.2. Safety Impact Analysis Based on GMM

Gaussian mixture model (GMM) is a probabilistic method often used for clustering and density estimation [32]. Since the residual characteristics of MPLs do not follow a normal distribution, traditional distance measures such as Mahalanobis distance cannot be directly applied. GMM can be used to decompose the non-Gaussian feature set into a combination of normal functions. GMM can be expressed as [33,34]:

(5) $$\begin{array}{l} p(x)=\sum_{i=1}^{m}w_{i}p_{i}(x)=\sum_{i=1}^{m}w_{i}N(x;u_{i},\sum_{i})\\ N(x;u_{i},\sum )=\frac{1}{{\left(2\pi \right)}^{n/2}|\sum {|}^{1/2}}e^{-\frac{1}{2}{\left(x-\mu \right)}^{T}\sum_{}^{-1}\left(x-\mu \right)} \end{array}$$

where m is the order of the mixture model; $w_{i}$ is the weight of the mixture model and satisfies; $\sum_{i=1}^{n}w_{i}=1;X=[x_{1},x_{2},\cdots ,x_{n}{]}^{T}$ is a n-dimensional vector; $u_{i}$ is the mean of the i Gauss model; and $\sum_{i}$ is a covariance matrix.

In this paper, the Kullback–Leibler (KL) divergence based on the best matched Gaussian component is used to calculate MI. Firstly, the KL divergence is defined based on Equation (6) [35], where $\Phi_{i}(0)$ represents the safe-state Gaussian components, and $\Phi_{j}(n)$ represents the unsafe-state Gaussian components. $\mu_{i}(0)$ and $\sum_{i}(0)$ are the parameters of Φ(0), while $\mu_{j}(0)$ and $\sum_{j}(0)$ are the parameters of Φ(n).

(6) $$D_{KL}(\Phi_{i}(0)||\Phi_{j}(n))=\frac{1}{2}\{tr[\Sigma_{j}{\left(n\right)}^{-1}\Sigma_{i}(0)-d-\ln[\frac{Det(\Sigma_{j}(n))}{Det(\Sigma_{i}(0))}]+{\left[\mu_{j}(n)-\mu_{i}(0)\right]}^{T}\Sigma_{j}{\left(n\right)}^{-1}[\mu_{j}(n)-\mu_{i}(0)]\}$$

If the minimum KL deviation is between $\Phi_{i}(0)$ and $\Phi_{j}(n)$, then in Φ(n), the best matching Gaussian component of $\Phi_{i}(0)$ is $\Phi_{j}(n)$. Based on this, it can find the best matching Gaussian component of the Gaussian component of each Φ(0) in Φ(n). The MI can be calculated with Equation (7).

(7) $$MI(\Phi (0),\Phi (n))=\sum_{i=1}^{C_{GMM}}\omega_{i}\underset{j}{\min}[D_{KL}(\Phi_{i}(0)||\Phi_{j}(n))+\ln\frac{\omega_{i}}{\omega_{j}}]$$

where $\omega_{i}$ and $\omega_{j}$ are the mixture weight of the two corresponding Gaussian components of Φ(0) and Φ(n), respectively.

## 4. Case Study

The physical failures and cyber-attacks are assumed as follows in the modeling process. The control goal of the whole system is to keep the cold plate temperature constant, and the temperature control point is located at the inlet of the cold plate. The attack on the control algorithm is not discussed at this time. The fault details are listed in Table 3. The simulation time is 6000 s, and the data sampling frequency is 100 Hz. Simulated faults/attacks were introduced at 3000 s. To train the ENN observer, the parameters of ENN are set as follows: the numbers of input neurons and hidden neurons are 5 and 10, respectively; the maximum training epoch is 200, and the expected error is 0.0001.

In the baseline system, the temperature at cold plate 1 ($T_{c1}$) shall be maintained at 37 °C to ensure the best performance of load operation. In the global controller, the control aim is the outlet flow of the main road, and the control variable is pump speed. In the local controller, the control aim is the cold plate temperature, and the control variable is the valve’s opening value.

For convenience, the following symbols’ meanings are shown in Table 4.

### 4.1. Normal State

**Figure 4:**
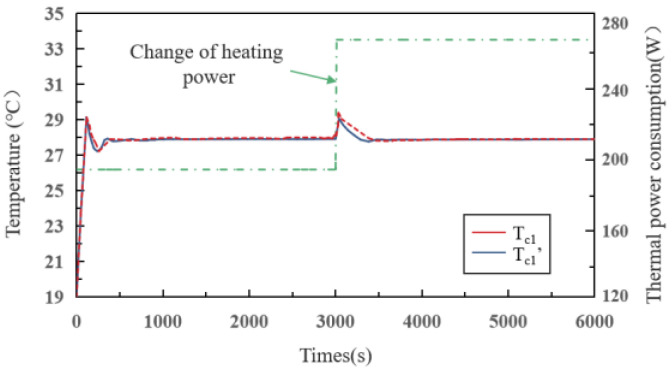
Comparison of simulated and actual temperature–time curves.

In order to verify the validity of the joint simulation model, we compared the simulation output results with the actual output results. The simulated outlet temperature (*T_c1′_*) and the actual outlet temperature of the cold plate 1 (*T_c1_*) are shown in Figure 4.

As can be seen, the overshoots of *T_c1′_* and *T_c1_* are almost the same. Before and after the heat source changes, the overshoots are 0.7 °C and 0.5 °C, respectively. Taken together, The Co-simulation model can accurately simulate the MPLs.

### 4.2. Case1: T Sensor Hard Failure

**Figure 5:**
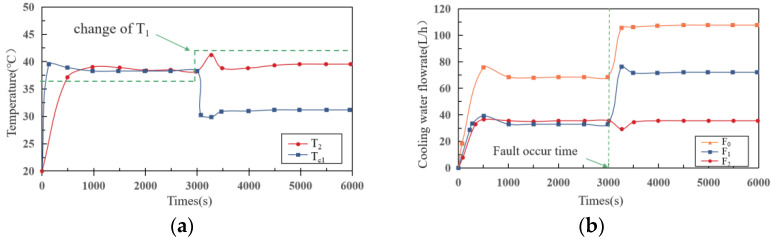
(**a**) Temperature–time curve; (**b**) flowrate–time curve in case 1.

In this scenario, we assume that the temperature sensor in branch 1 has failed, resulting in a step-change in the value of *T*_1_. During the fail time from 3000 s, we corrupted the number of *T*_1_ from 37 °C to 43 °C. The system response under failure was simulated and evaluated using the proposed framework. Figure 5 compares the MPLs system’s response under failure-free and failure-injected situations. There are two main concerns: the actual temperature of the cold plate and the flow of each branch. In the first stage, under the action of closed-loop control, all temperatures rise rapidly and stabilize after about 500s. The temperature of coldplant in branch 1 (*T_c1_*) is basically the same as the temperature of T sensor in branch 2 (*T*_2_*)* after reaching a steady state, about 37.2 °C. In the second stage, the value of *T*_1_ jumps from 37 °C to 43 °C, resulting in a rapid reduction in *T_c1_*. Furthermore, there is a slight increase in *T*_2_, which returns to a steady state after 4000 s.

The main reason for the above phenomenon is the change in the valve opening and flow rate of branch 1. When the fault occurs, to reduce the temperature of *T*_1_ to the specified range, the local controller increases the opening valve of branch 1, thus increasing the flow in branch 1. Since the pump speed and main road pressure are both constant, the increase in the flow rate of branch 1 will correspondingly reduce the flow rate of branch 2. The reduction in the flow of branch 2 further causes *T*_2_ to decrease, which then triggers the adjustment of the flow valve of branch 2 until *T*_2_ returns to within the specified range.

### 4.3. Case2: F Sensor Soft Failure

**Figure 6:**
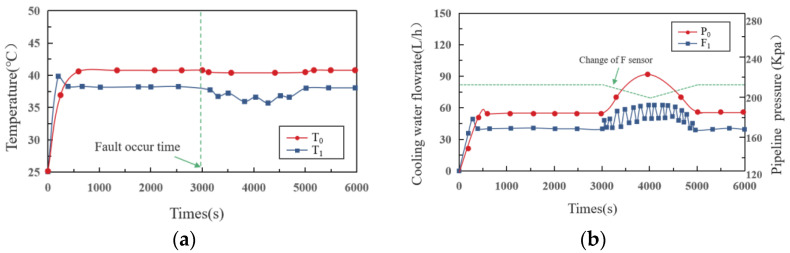
(**a**) Temperature–time curve; (**b**) flowrate–time curve in case 2.

In this scenario, we assume that the flow sensor in the main road has failed, resulting in a slow change in value. During the fail time, we gradually reduce the value of the sensor from 87 L/h to 75 L/h before resetting it to 87 L/h. As shown in Figure 6a, *T*_0_ and *T*_1_ are the main concerns in case 2. It should be pointed out that in this paper, since the working conditions of branch 1 and branch 2 are the same, only the relevant temperature of *T*_1_ is shown in the figure. Similar to case 1, in the first stage, under closed-loop control, the temperatures rise rapidly for about 500 s before stabilizing. The maximum values of *T*_0_ and *T*_1_ are 42 °C and 38 °C, which means that the temperature variations between the main road and the branch road is not significant. In the second stage, with the continuous decrease in flow, a constant oscillation in the *T*_1_ value is traced by a trend of first decreasing, then increasing. The value of *T*_1_ is finally stabilized at 41.3 °C after a slight decrease and attains a stable state soon after.

The main reason for the above phenomenon is the change in valve opening and pump speed, as shown in Figure 6b. When fault occurs, firstly, the circulating pump reacts quickly and increases its speed to maintain the corresponding flow of the main road. This leads to a corresponding increase in the flow of the two branches, leading to a decrease in *T*_1_. After the temperature drops, the valve adjustment will be triggered to ensure a stable temperature at the cold plate. This repeated adjustment will cause the fluid in the pipeline to oscillate, and the pressure will continue to rise until the end of the fault.

### 4.4. Case 3: Cyber-Attacks

**Figure 7:**
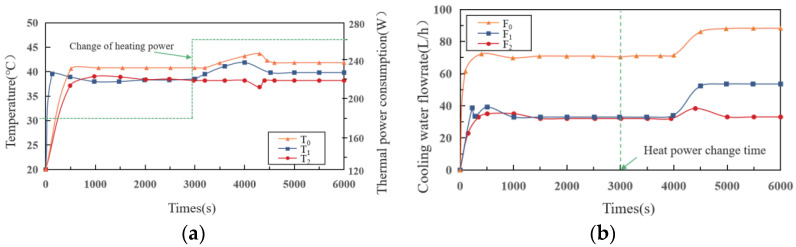
(**a**) Temperature–time curve; (**b**) flowrate–time curve in case 3.

In this scenario, we simulate a DoS attack that happened on the transmission of variable setpoint from the controller to the valve. The DoS attack will block the valve from receiving the setpoint reset signals. Before the attack occurs, the temperature change of each branch is consistent with the previous two cases, which will not be described in this section. When the attack occurs, if the heat source remains unchanged, although the attack blocks the signal transmission, the valve opening can be maintained in the state of the previous time. Obviously, the attack has no impact on the system. Nevertheless, when the heat source changes, DoS attack impacts the system’s stable operation. As shown in Figure 7, when the heat source changes from 180 W to 260 W, *T*_1_ and *T*_0_ rise gradually, while *T*_2_ remains unchanged, where the temperature change time of *T*_0_ is approximately 100 s later than *T*_1_. After 4000 s, the values of *T*_1_ and *T*_0_ start to decrease, and the rate of decline is higher than the rate of increase.

Since the valve opening of branch 1 cannot be adjusted, the increase in heat source will lead to an increase in *T*_1_. Due to the large specific heat capacity of the cold plate, the value of *T*_1_ rises gradually. Affected by the rise in *T*_1_, *T*_0_ also gradually increases. Then, the global control is started to increase the speed of pump, which results in the increased flow of each branch. Under the action of valve regulation, the flow of branch 2 returns to a steady state.

### 4.5. Impact Analysis of the Three Cases

**Figure 8:**
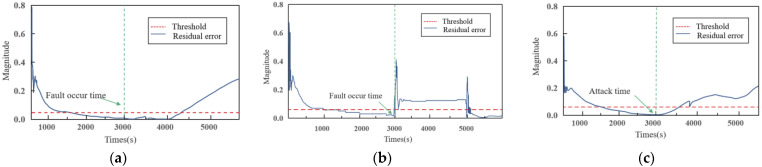
Residual errors (**a**) Case 1; (**b**) Case 2; (**c**) Case 3.

Figure 8 compares the residual errors of the above three cases. The figure shows that there are short-term fluctuations in the residuals during t = 0 s~200 s due to errors between the actual and estimated values, which can be ignored during the fault analysis. For the overall system, residuals are close to zero whenever the systems run normally, and residuals increase significantly when faults occur. The observers proposed in this paper can effectively track the status. It can be seen that, in case 1 and case 2, when a fault occurs, the residual errors respond quickly to exceed the thresholds, showing a strong sensitivity, while in case 3, the residual errors exceed the threshold after a certain time delay. This reflects that the observers proposed in this paper are more sensitive to physical failures than cyber-attacks.

**Figure 9:**
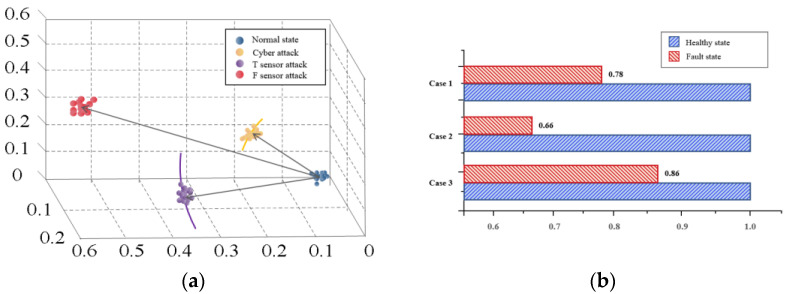
(**a**) Demonstration of baseline GMM and unsafe-status GMM; (**b**) the safe state analysis results of the proposed method.

The MI, which represents system safety state, is shown in Figure 9.

Comparing case 1 and case 2, although both sensors failed, the MI of case 1 is 0.78, which is larger than the MI of case 2 (0.66). In case 1, the system can recover to a stable state quickly after failure. In case 2, once failure occurs, the temperature and flow values repeatedly fluctuate; in particular, the surge of pipeline pressure seriously impacts the system operation. Except for different failure modes, the T sensor participates in the local control strategy, while the F sensor participates in the global control strategy is also an important reason.

Comparing case 1 and case 3, the MI of case 1 is 0.86, while that of case 2 is 0.78. The MI difference between the two is the smallest. Through a comparison, it is obvious that the impact of a DoS attack is smaller than that of a T sensor failure. On the other hand, in the first stage of case 3, the slow change in the T sensor can be regarded as a soft failure; the system takes nearly 2000 s to recover to the steady-state. Contrastingly, in case 1, the system can recover to a stable state in about 500 s after the hard failure of T sensor. Therefore, the impact of hard failure (step change) is also smaller than that of soft failure (slow change).

Comparing case 2 and case 3, the MI difference is the largest. Sensor failure and DoS attacks could drive the system to an unsafe state. Compared with DoS attacks, sensor failures have a greater impact on the health of the whole system. However, considering that the time of fault detection in case 3 is much larger than that in case 1, it cannot be assumed that the risk of DoS attack is lower than that of sensor failure.

## 5. Conclusions

This paper presents a joint simulation and modeling approach for analyzing the impact of physical faults and cyber-attacks on system operation. The framework discusses three common attack and failure modes: sensor hard failure that occurs suddenly, sensor soft failure that occurs gradually over time, and DoS attacks that prevent communication between the controller and valve. The safety state of MPLs under different failure and attack modes are quantified by ENN observers and GMM-KL algorithms, which can standardize and rank the severity. The results show that both sensor failures and DoS attacks render the system unsafe, according to simulation data. In comparison to DoS attacks, however, sensor failures, particularly soft failures, inflict the greatest harm to the MPLs. Therefore, it is crucial to apply multi-sensor information fusion techniques in the control of MPLs to mitigate the potential impacts caused by single sensor failures. Furthermore, sensors engaged in global control, rather than those involved in local control, need additional protection. The system control strategy should prioritise temperature control rather than flow control.

This technique may assist designers in comprehending the effects of various failures and assaults on MPLs, allowing them to utilize limited resources while building and prioritizing defense tactics, hence facilitating safety evaluations in the engineering area.

Work to be conducted in the future includes: (1) testing the method with more comprehensive failure and attack modes, especially combined or multiple failure modes. (2) Using more parameters to evaluate more complex systems, e.g., consider increasing heat transfer efficiency of heat exchangers, line flow resistance, etc. in the simulation modeling. (3) Employing other intelligent algorithms to improve the accuracy of evaluation, such as Convolutional Neural Network (CNN), Recurrent Neural Network (RNN), etc.

## Figures and Tables

**Table 1:** The Main Simulation Parameters.

Parameters	Values
Initial temperature	30 °C
Initial flowrate	70 L/h
Pump speed	150 rev/min
Pipe diameter	8 mm

**Table 2:** The Main Simulation Parameters.

Target	Failure/ Attack	Failure/Attack Modeling	Mathmatical Formulations
Sensors	Hard failure	Add a fix bias in the output value	$\hat{y}(t)=\{\frac{y(t),t\notin A}{y(t)+Q,t\in A}$
	Soft failure	Add a recoverable interference to the output value	$\hat{y}(t)=\{\frac{y(t),t\notin A}{y(t)+f_{p}(t),t\in A}$
network	Denial of service	Make the actuator continuously receive the instruction of the last time	$\hat{u}(t)=\{\frac{u(t),t\notin A}{u(t-1),t\in A}$

**Table 3:** Test details for fault/attack simulation.

No.	Failure/ Attack Mode	Changed Parameter for Simulation	Parameter (Normal)	Parameter (Abnormal)
Case1	T sensor failure	Signal output	37 °C	43 °C
Case2	F sensor failure	Signal output	87 L/h	87 L/h~75 L/h~87 L/h
Case3	DoS attack	Communication rate	100%	0%

**Table 4:** Symbol meanings.

Symbols	Meaning		
*T_0_*	The outlet temperature of the main road	*F_0_*	The outlet flow of main road
*T_1_*	The temperature of T sensor in branch 1	*F_1_*	The flowrate of F sensor in branch 1
*T_2_*	The temperature of T sensor in branch 2	*F_2_*	The flowrate of F sensor in branch 2
*T_c1_*:	The temperature of coldplant in branch 1	*P_t_*:	The pressure of main road

## Data Availability

Data available on request from the authors.
